# Error

**Published:** 1854-06

**Authors:** 


☞ An error has been discovered, too late for correction, in the paging of this number. The pages should be numbered progressively from page 328 to page 345.

